# Meta-analysis of the effect of PGM on survival prognosis of tumor patients

**DOI:** 10.3389/fonc.2022.1060372

**Published:** 2022-12-05

**Authors:** Zhewen Zheng, Jian Bai, Shuangting Shen, Chunmei Zhu, Yunfeng Zhou, Xue Zhang

**Affiliations:** ^1^ National Cancer Center/National Clinical Research Center for Cancer/Cancer Hospital & Shenzhen Hospital, Chinese Academy of Medical Sciences and Peking Union Medical College, Shenzhen, China; ^2^ Department of General, Surgery, Xuanwu Hospital Capital Medical University, Beijing, China; ^3^ District Central Hospital, Nanjing, Jiangsu, China; ^4^ Department of Radiation Oncology and Medical Oncology, Zhongnan Hospital of Wuhan University, Wuhan, Hubei, China; ^5^ Department of General Practice, Beijing Friendship Hospital, Capital Medical University, Beijing, China

**Keywords:** cancer, PGM, survival prognosis, glycogen metabolism, meta-analysis

## Abstract

**Objective:**

A systematic evaluation of the impact of phosphoglucose translocase PGM on the survival prognosis of tumor patients was conducted to understand its impact on tumors so as to improve the quality of survival and to find effective therapeutic targets for tumor patients.

**Methods:**

The following were searched in the databases China National Knowledge Infrastructure (CNKI), Wanfang, Wipu, PubMed, EMBASE, ScienceDirect, Web of Science, and Cochrane Library: “PGM1”, “PGM2”, “PGM3”, “PGM4”, and “PGM5” as Chinese keywords and “PGM1”, “PGM2”, “PGM3”, “PGM4”, “PGM5”, “PGM1 cancer”, “PGM2 cancer”, “PGM3 cancer”, “PGM4 cancer”, “PGM5 cancer”, and “phosphoglucomutase”. Relevant studies published from the database establishment to April 2022 were collected. Studies that met the inclusion criteria were extracted and evaluated for quality with reference to the Cochrane 5.1.0 systematic evaluation method, and quality assessment was performed using RevMan 5.3 software.

**Results:**

The final results of nine articles and 10 studies with a total of 3,806 patients were included, including 272 patients in the PGM1 group, 541 patients in the PGM2 group, 1,775 patients in the PGM3 group, and 1,585 patients in the PGM5 group. *Results of the meta-analysis*: after determining the results of the nine articles, it was found that the difference was statistically significant with a p-value <0.05 (hazard ratio (HR) = 0.89, 95% CI 0.69–1.09, p = 0.000). To find the sources of heterogeneity, the remaining eight papers were tested after removing the highly sensitive literature, and the results showed I^2^ = 26.5%, p < 0.001, a statistically significant difference. The HR for high expression of PGM1 and PGM2 and PGM5 was <1, while the HR for high expression of PGM3 was >1.

**Conclusion:**

Although PGM1, PGM2, PGM3, and PGM5 are enzymes of the same family, their effects on tumors are different. High expression of PGM1, PGM2, and PGM5 can effectively prolong the overall survival of patients. In contrast, high expression of PGM3 reduced the overall survival of patients. This study of PGM family enzymes can assist in subsequent tumor diagnosis, treatment, and prognostic assessment.

## Introduction

Phosphoglucomutase (PGM), a key enzyme involved in the synthesis and breakdown of glycogen, is essential in the formation of carbohydrates from G-6-P and the formation of G-6-P from galactose and glycogen (1). At this stage, five enzymes of the PGM family have been identified: PGM1, PGM2, PGM3, PGM4, and PGM5. The coding sequences of these five enzymes are homologous, but due to their different substrates and functions, their effects on tumors are also different (2). This study found that PGM is basically involved in glucose metabolism (3, 4). PGM2 expression levels may affect the reduction of glucose-1,6-bisphosphate expression levels in human erythrocytes (5). PGM3 is targeted to inhibit the hexosamine biosynthesis pathway, inhibit tumor growth, and promote apoptosis (6). PGM4 has been less studied, and no in-depth studies have been found in the literature. Mutations in PGM1 gene cause PGM1 deficiency, which is classified as an inborn metabolic disorder and was once identified as a glycogen accumulation disorder. Existing studies have found that PGM1 deficiency is a congenital glycosylation disorder (7–9). An increasing number of studies have found that PGM1 influences tumor development through its involvement in glycogen metabolic processes (10, 11). PGM5 has been studied mainly in muscle tissue and is highly expressed in cardiac muscle, skeletal muscle, and smooth muscle (12). It is mainly distributed in the periphery of myofibroblasts, localized at intercellular adhesion junctions, and plays an important role in cell adhesion junctions and cytoskeleton maintenance. Available studies have shown that PGM5 is important for the diagnosis and prognosis of a variety of tumors (13, 14). At this stage, there are many ways to treat tumors, such as surgery, chemotherapy, and radiotherapy. However, the efficacy of these treatments is not significant for patients with advanced stages. In order to improve the survival quality of tumor patients and find effective therapeutic targets, it is necessary to study the effect of PGM on the survival prognosis of tumor patients.

In this review, we conducted a meta-analysis of the data related to PGM homologous enzymes affecting the survival prognosis of tumor patients to understand the role of PGM family-related enzymes in tumors and to assess the risk of PGM1, PGM2, PGM3, and PGM5 on survival prognosis so as to provide more evidence-based medical evidence for clinical treatment and prognosis judgment.

## Methods

### Data sources

The following were searched in the databases China National Knowledge Infrastructure (CNKI), Wanfang, Wipu, PubMed, EMBASE, ScienceDirect, Web of Science, and Cochrane Library: “PGM1”, “PGM2”, “PGM3”, “PGM4”, and “PGM5” as Chinese keywords and “PGM1”, “PGM2”, “PGM3”, “PGM4”, “PGM5”, “PGM1 cancer”, “PGM2 cancer”, “PGM3 cancer”, “PGM4 cancer”, “PGM5 cancer”, and “phosphoglucomutase”. Relevant studies published from the database establishment to April 2022 were collected.

### Study selection

Inclusion criteria: 1) PGM homologous enzymes (PGM1, PGM2, PGM3, PGM4, and PGM5), 2) OS data are included in all literature, 3) large data sample size, and 4) clear and complete data sources. Exclusion criteria: 1) missing data for the study, e.g., number of missing pieces; 2) incomplete information and incomplete research; 3) duplicate publication; 4) overviews, case reports, etc.; 5) unclear efficacy evaluation; and 6) excluded articles related to research on anoxic microenvironment.

### Data extraction

On the basis of the inclusion and exclusion criteria, two evaluators independently conducted database searches and screened the literature, and differences in opinion, when encountered, were resolved through discussion. Information extraction was performed for the final included literature: 1) basic information of the literature: title, author, time of publication, and country or region; 2) clinical study information: type of study, number of cases, and treatment protocol; and 3) outcome indicators: if two evaluators could not agree, a third evaluator was asked to participate in the decision.

### Quality evaluation

Quality assessment of the included literature was performed using the *Cochrane Handbook for Systematic Reviews of Interventions*.

### Statistical analysis

The OS data plots for each group were first analyzed using Engauge Digitizer software, and the final hazard ratio (HR) and 95% CI were obtained using the tool of Jayne F. Tierney et al. The quality of the literature was evaluated using RevMan 5.3 software, and a meta-analysis of all the outcome indicators of the included studies was performed using Stata 12 software, using HR and its 95% CI as effect evaluation criteria. Statistical heterogeneity of the literature was analyzed using I^2^, and when p ≥ 0.05 and I^2^ ≤ 50%, studies were considered to have no statistical heterogeneity, and fixed-effects models were used. When p < 0.05 and I^2^ > 50%, studies were considered to have statistical heterogeneity, random-effects models were used, and the sources of heterogeneity were further discussed.

## Results

### Study selection

**Figure 1:**
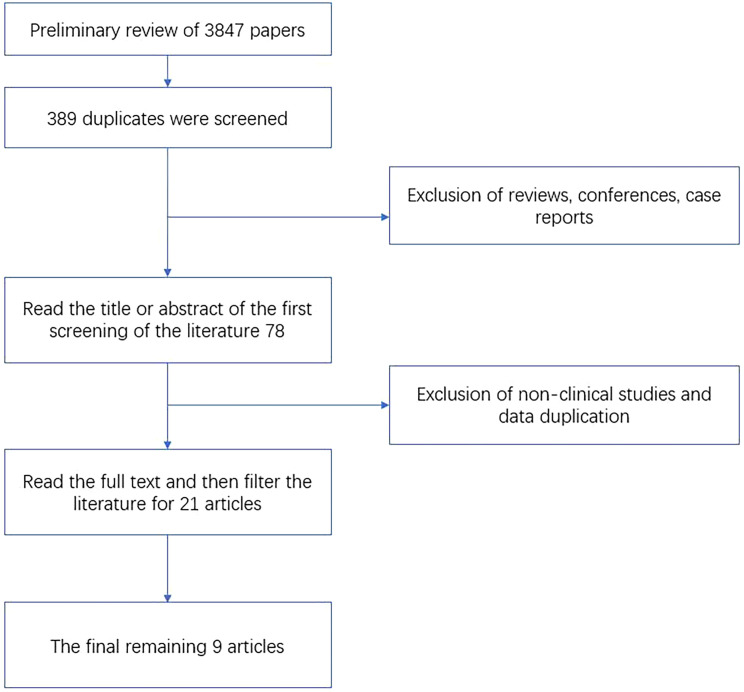
Flow chart of literature screening.

According to the search strategy, a total of 3,847 relevant articles were obtained for the initial screening. Duplicates were removed, and reviews, conferences, and case reports were excluded. The titles, abstracts, and full texts were further read; 21 were re-screened by combining the inclusion and exclusion criteria. There was 0 article on PGM4, and nine articles finally met the criteria. The literature screening process and results are shown in **Figure 1**.

### General characteristics

Ten studies from nine included papers had a total of 3,806 patients, including 272 in the PGM1 group, 446 in the PGM2 group, 1,503 in the PGM3 group, and 1,585 in the PGM5 group. The basic characteristics of the nine papers were analyzed and are shown in **Table 1**.

**Table 1:** Basic characteristics of the included studies.

Author	Year	Country	Related factors	Tumor type	Data source	Survival analysis	n	HR (95% CI)
Guang-Zhi Jin, et al. (10)	2018	China	PGM1	Liver cancer	Non-database	OS/CIR	272/272	0.75 (0.45–1.24)
Zhongqi Cui, et al. (15)	2021	China	PGM2	Colon cancer	TCGA	OS	446	0.54 (0.37–0.79)
Francesca Ricciardiello, et al. (16)	2020	Italy	PGM3	Pancreatic cancer	Non-database	OS	95	1.89 (0.75–4.77)
Hyunmin Lee, et al. (17)	2022	United States	PGM3	Bladder cancer/breast cancer	OncoLnc	OS	402/1006	1.3 (1–1.7) 1.35 (1.09–1.66)
Yan Jiao, et al. (14)	2019	China	PGM5	Liver cancer	TCGA	OS/CIR	367/320	0.82 (0.61–1.11)
Yifan Sun, et al. (13)	2019	China	PGM5	Colon cancer	TCGA	OS	79	0.79 (0.27–2.29)
Zaizai Cao, et al. (18)	2020	China	PGM5	Oral cancer	TCGA	OS	516	0.93 (0.70–1.11)
Bing Chen, et al. (19)	2021	China	PGM5	Lung adenocarcinoma	TCGA	OS	478	0.65 (0.55–0.88)
Fang Ran, et al. (20)	2021	China	PGM5	Breast cancer	METABRIC	OS/DFS	145/145	0.64 (0.19–2.11)

OS, overall survival; DFS, disease-free survival; CIR, cumulative incidence of relapse; HR, hazard ratio.

### Methodological quality evaluation

**Figure 2:**
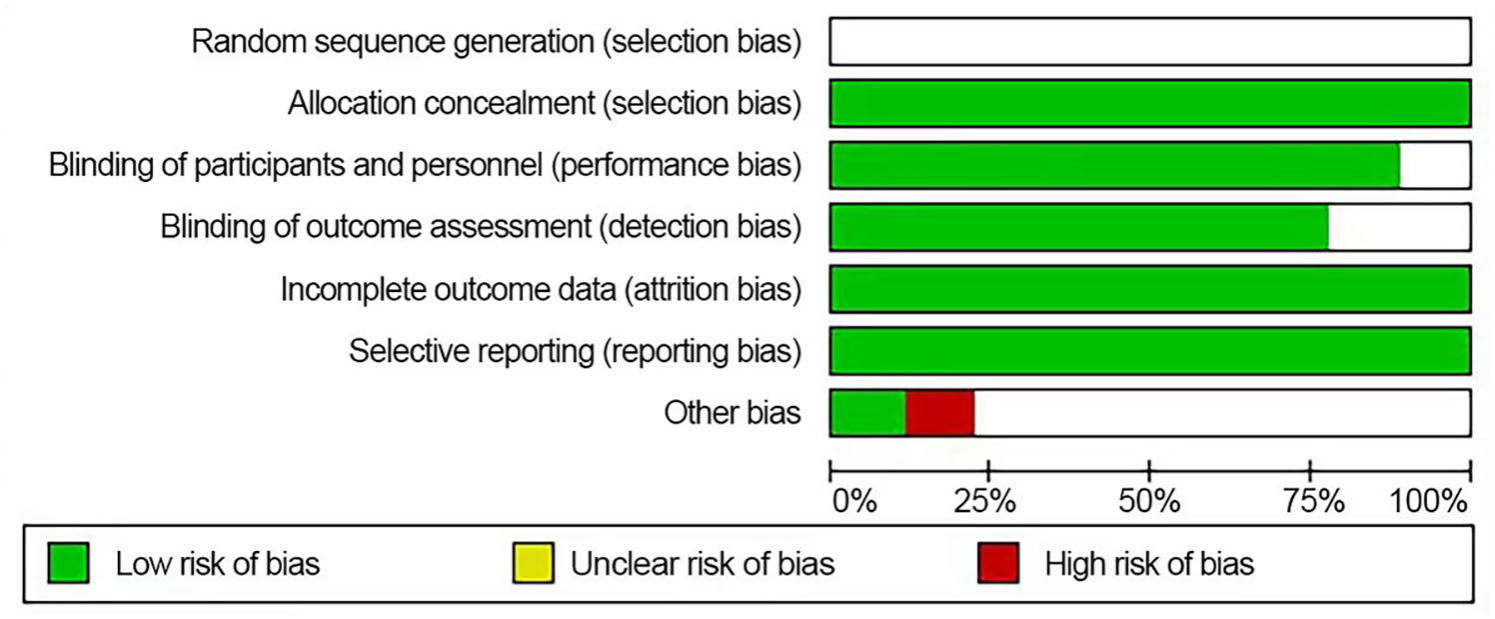
The form of literature quality evaluation.

The quality evaluation and risk assessment of the included literature are shown in **Figure 2**.

### Meta results

#### Effect of PGM homologous enzymes on hazard ratio

**Figure 3:**
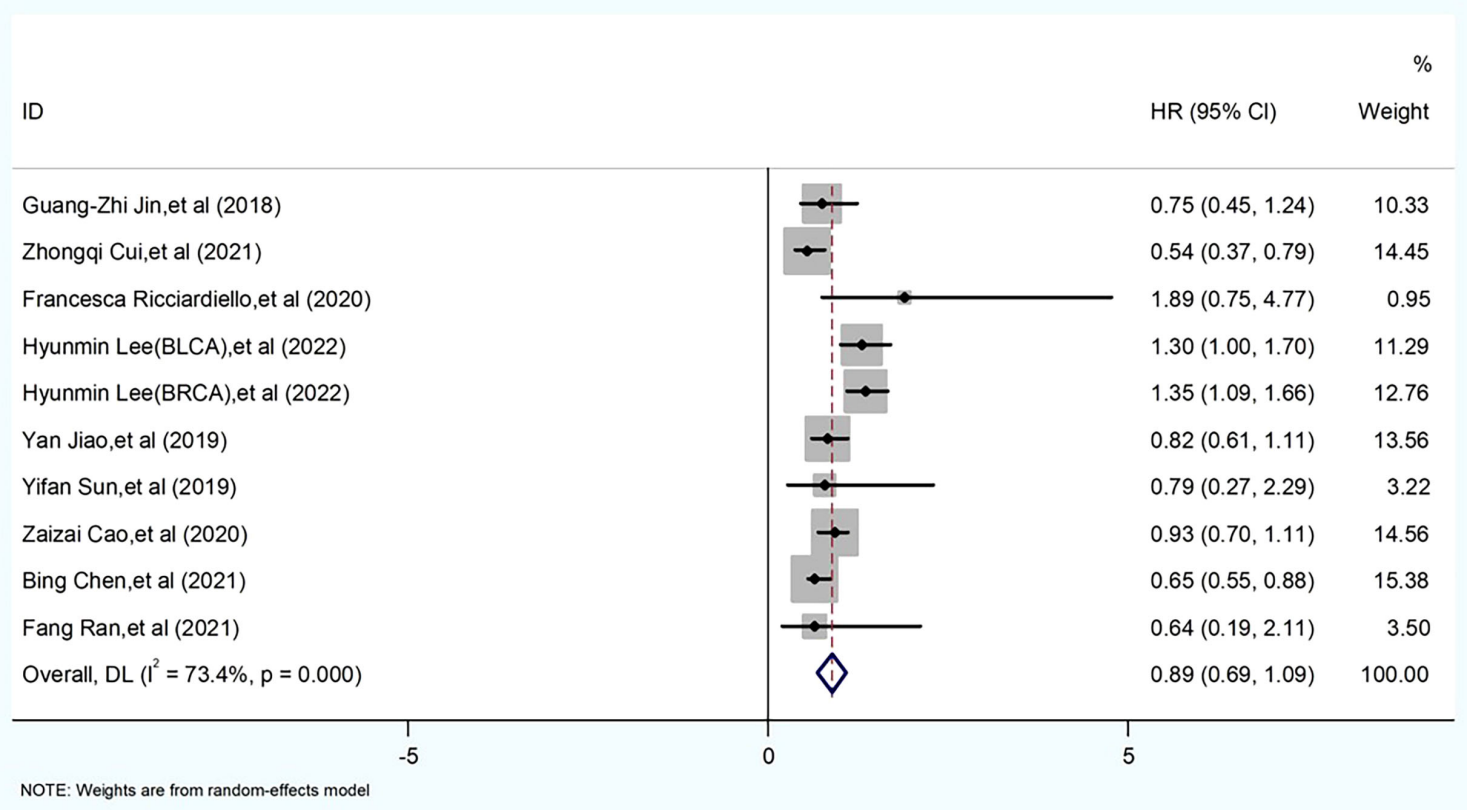
Meta-analysis of PGM homologous protein expression profile and survival prognostic risk ratio (HR) in forest plot.

In this included literature, 10 data sets from nine papers were statistically analyzed. Forest plot analysis revealed a mixed picture of the effect of PGM homologous enzymes on tumor cells. *Evaluation criteria*: the impact of the expression of PGM homologous enzymes in tumor patient tissues on patient survival prognosis was examined. A heterogeneity test was performed on these 10 groups of data, I^2^ = 73.4%, p = 0.000, and a meta-analysis was performed using a random-effects model because the I^2^ value was >50%. The results showed that the HR for high expression of PGM1 and PGM2 and PGM5 was <1, while the HR for high expression of PGM3 was >1. The difference was statistically significant (HR = 0.89, 95% CI 0.69–1.09, p < 0.001) (**Figure 3**). Thus, high expression of PGM1, PGM2, and PGM5 inhibited tumor development. High expression of PGM3 promoted tumor development.

### Sensitivity analysis

**Figure 4:**
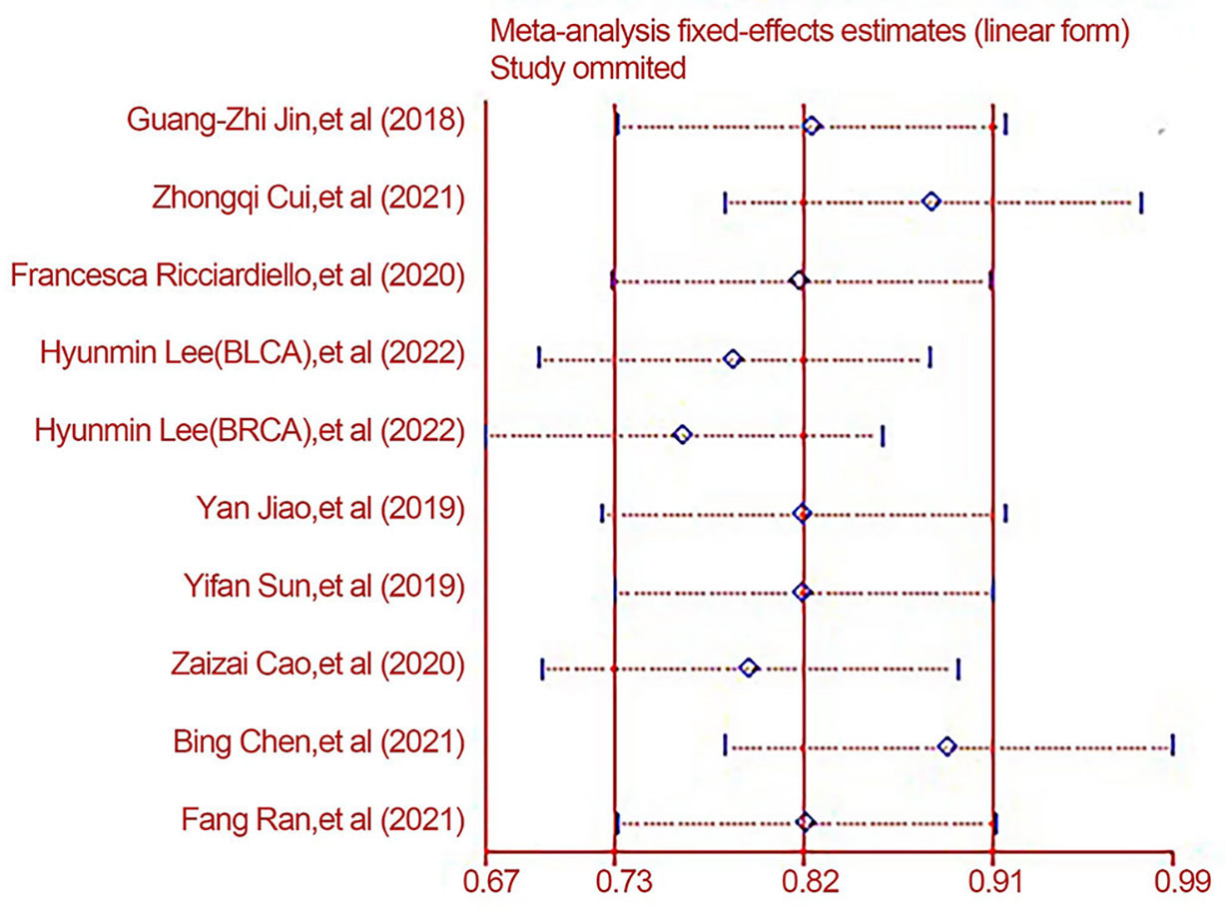
The Overall Sensitivity analysis between different groups.

**Figure 5:**
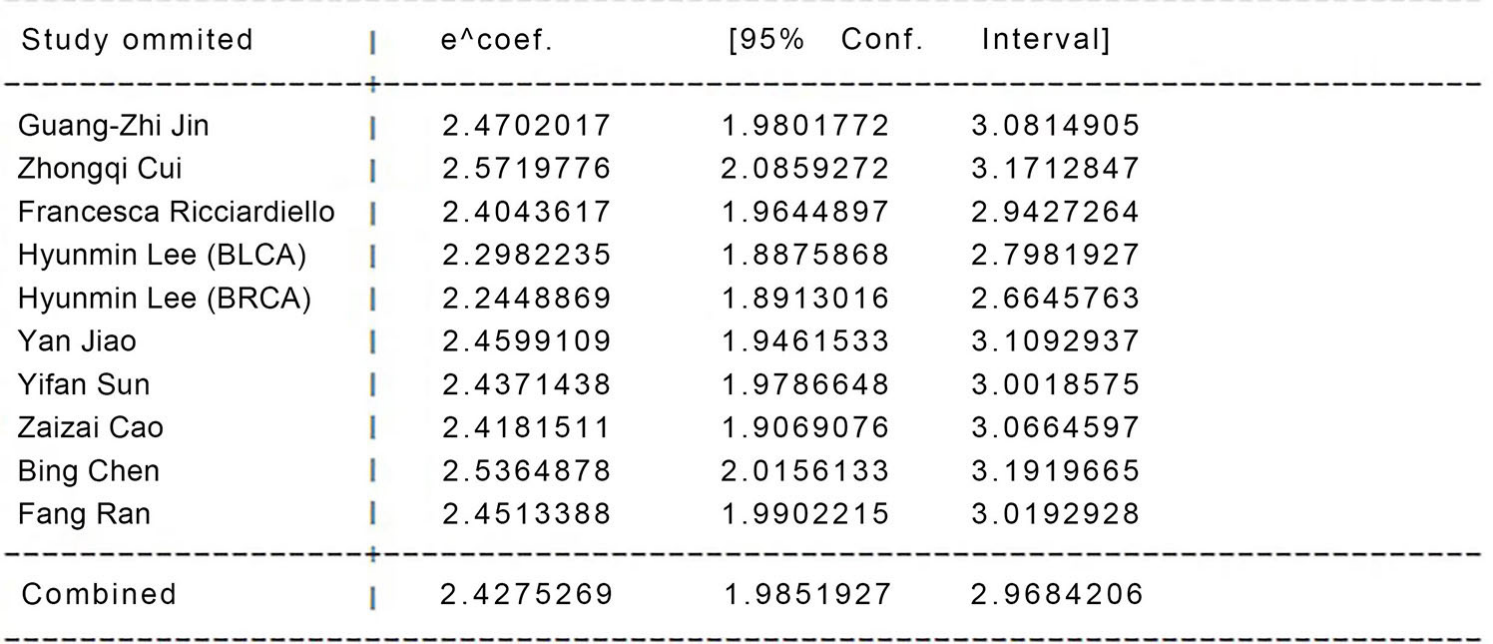
Specific data for sensitivity analysis between groups to the overall.

As we can see from **Figures 4**. **5**, almost all studies’ combined effect sizes were within the 95% CI. However, by comparing the specific data through sensitivity analysis, we can see that the three reports by authors Hyunmin Lee, Zhongqi Cui, and Bing Chen have the potential to create heterogeneity in the overall data (**Figures 4**, **5**).

### Data analysis (after excluding heterogeneity)

**Figure 6:**
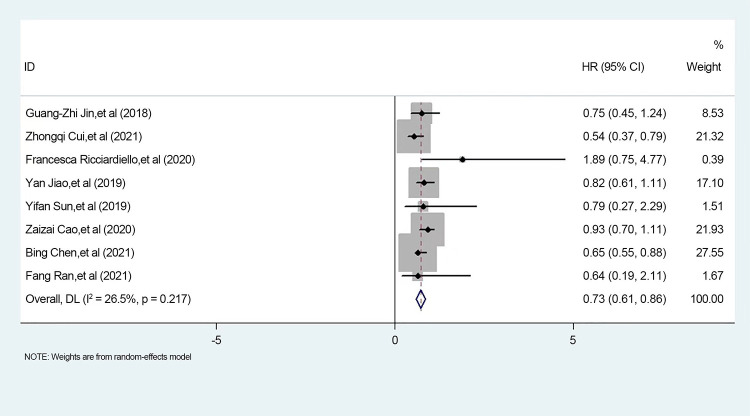
Meta-analysis of PGM homologous protein expression profile and survival prognostic risk ratio (HR) in forest plot.

We found that the literature by Lee et al. was responsible for the elevated heterogeneity, and removing the literature had the potential to cause elevated heterogeneity separately in order to clarify the source of heterogeneity. After we removed this literature, we performed heterogeneity tests on the remaining eight papers and found that I^2^ = 26.5%, p < 0.001, a statistically significant difference. Since the I^2^ value was <50%, we made forest plots using a fixed-effects model (**Figure 6**). The results showed that high expression of PGM1 and PGM2 and PGM5 inhibited tumor development and that high expression of PGM3 promoted tumor development.

### Publication bias

**Figure 7:**
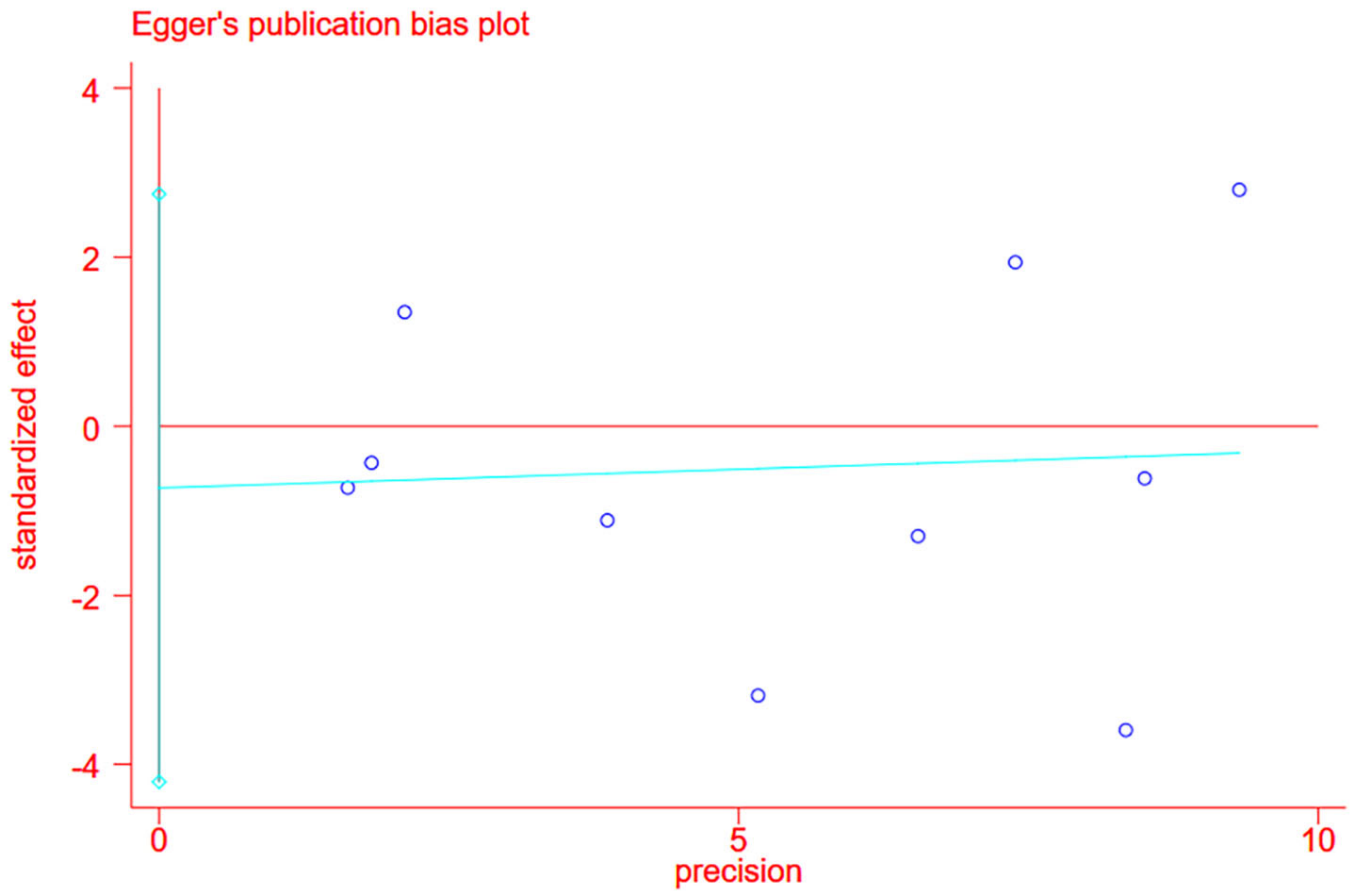
The plot of literature publication offset funnel.

Publication bias was evaluated using Stata 12 software, and Egger’s test was used for evaluation. To ensure the effectiveness of the funnel plot test, nine groups of data were included, one of which analyzed the survival prognosis of two groups of tumors. Therefore, a total of 10 groups of data were included for analysis. The possibility of bias was determined by funnel plot with p-value = 0.641, p > 0.05, indicating no publication bias (**Figure 7**).

## Discussion

Tumor is one of the major diseases that people need to overcome in the 21st century. Through the meta-analysis of PGM1, PGM2, PGM3, and PGM5 in PGM homologous enzymes, we hope to understand the role of PGM homologous enzymes in tumors and provide assistance for subsequent treatment and research.

PGM1 is an important key enzyme in the processes of glycogen synthesis and catabolism, which catalyzes the reversible transfer of phosphate at the α-d-glucose 1 and 6 positions. On the one hand, the G-1-P produced by glycogen catabolism is transformed into the first intermediate product G-6-P in the glycolysis process. On the other hand, G-6-P is converted into G-1-P to produce the substrate for the synthesis of uridine diphosphate glucose (UDP-glucose), which is necessary for the synthesis of many cellular components such as glycoproteins (21, 22). PGM1 inhibits cell proliferation and tumor growth by utilizing sufficient extracellular glucose for conversion to glycogen in an aerobic environment, while its deletion inhibits glycogen synthesis, leading to more glucose for glycolysis, which promotes tumor cell proliferation and tumor growth. Jin et al. (10)showed that PGM1 could inhibit the progression of hepatocellular carcinoma by regulating glucose transport. Therefore, the high expression of PGM1 can inhibit the development of tumors.

Phosphoglucomutase 2 (PGM2) also catalyzes the reversible conversion of glucose 1-phosphate to glucose 6-phosphate. PGM2 has been reported to be a biomarker for potential prognostic assessment of renal clear cell carcinoma (23). The study by Yang et al. (24) illustrated that PGM2 can be an important indicator for the prognosis of colorectal cancer. The high expression of PGM2 can inhibit the development of tumors.

PGM3 is a member of the hexose-phosphate metastable enzyme family and plays a major role in glycogenolysis and gluconeogenesis. PGM3 is a *N*-acetylglucosamine triphosphatase involved in the biosynthesis of aminoalanine, which exerts anti-cancer effects (25). Lee et al. (17) showed that targeted therapy against PGM3 could be a therapeutic strategy for KRAS/LKB1 co-mutant lung cancer, validating the tumor-promoting effect of high expression of PGM3.

PGM5, also known as phosphoglycosidase-related protein (PGM-rp) or aciculin, is located on human chromosome 9 (9q21.11) (26). PGM5 shows high expression in smooth muscle, skeletal muscle, and cardiac muscle. PGM5 also has an important effect on glycolysis, and several studies have reported that high expression of PGM5 inhibits tumor development. Ran et al. (20) showed that miR-1224-3p promotes the proliferation and migration of breast cancer cells through PGM5-mediated aerobic glycolysis. Sun et al. (13) showed that PGM5 predicted the prognosis of colorectal cancer patients. Jiao et al. (14) showed that PGM5 is a biomarker for the diagnosis and prognosis of hepatocellular carcinoma.

In this paper, we included nine articles and 10 study results with a total of 3,806 patients, including 272 patients in the PGM1 group, 446 patients in the PGM2 group, 1,503 patients in the PGM3 group, and 1,585 patients in the PGM5 group. Through meta-analysis, we found that when PGM1, PGM2, and PGM5 are highly expressed, their HR is less than 1, indicating that PGM1, PGM2, and PGM5 can improve the overall survival rate of patients and have an inhibitory effect on tumors. When PGM3, which is also a PGM homologous enzyme, is overexpressed, its HR is >1, indicating that the overexpression of PGM3 will reduce the overall survival rate of patients and play a role in the development of tumors. Therefore, it is necessary to study PGM to assist in follow-up treatment and prognosis judgment.

The main reason for the high heterogeneity of the studies included in this paper may be related, first of all, to the expression of PGM between different tumors. The tumors selected for this experiment were different among the PGM isoenzymes, which may be one of the reasons for this high heterogeneity. Even the same tumor may have a different expression for different PGMs. In this experiment, the effect of PGM3 on tumors was opposite to that of PGM1, PGM2, and PGM5, which may be the reason for the increased heterogeneity in the literature by Lee et al.

This study has several limitations. 1) The number of included studies was too small for corresponding subgroup analysis. 2) Bias exists despite a comprehensive search.

## Conclusion

In this study, based on the available clinical data, we performed a meta-analysis of the data on the expression of PGM homologous enzymes affecting the survival prognosis of tumor patients. The results showed that although PGM1, PGM2, PGM3, and PGM5 are homologous enzymes, their effects on tumors are different. The high expression of PGM1, PGM2, and PGM5 effectively prolonged the survival of patients. In contrast, high expression of PGM3 decreased the survival of patients. We hope that the meta-analysis can provide some reference for the study of clinical treatment and targeted drug therapy.

## Data availability statement

The original contributions presented in the study are included in the article/supplementary material. Further inquiries can be directed to the corresponding author.

## Author contributions

Methodology: ZZ, XZ, JB, and YZ. Software: XZ, ZZ, and CZ. Validation: ZZ and SS. Writing—original draft preparation: XZ and ZZ. Supervision: XZ, ZZ, and YZ. Project administration: XZ, ZZ, and YZ. All authors contributed to the article and approved the submitted version.

## Funding

This study was financially supported by the National Natural Science Foundation of China (81472799).

## Acknowledgments

We would like to acknowledge the reviewers for their helpful comments on this paper.

## Conflict of interest

The authors declare that the research was conducted in the absence of any commercial or financial relationships that could be construed as a potential conflict of interest.

## Publisher’s note

All claims expressed in this article are solely those of the authors and do not necessarily represent those of their affiliated organizations, or those of the publisher, the editors and the reviewers. Any product that may be evaluated in this article, or claim that may be made by its manufacturer, is not guaranteed or endorsed by the publisher.
